# Clinical Characteristics and Outcomes of Malaria Patients in the Aseer Region, Saudi Arabia: A Retrospective Study (2022–2025)

**DOI:** 10.3390/tropicalmed11040108

**Published:** 2026-04-20

**Authors:** Fouad Ibrahim Alshehri, Dhaifullah Ahmed Alkhosafi, Essam Abdullah Al Asmari, Abdulrahman Bin Saeed, Anas Mohammed Zarbah, Saeed Ali Algarni, Mohammed Gasim Ahmed, Marim Abdallah Mohamed, Fatma Anter Mady, Saleh Mohammed Zafer Albakri, Ramy Mohamed Ghazy

**Affiliations:** 1 Internal Medicine Department, Khamis Mushayt Hospital, Khamis Mushait 62461, Saudi Arabia; fialshehri@moh.gov.sa (F.I.A.); al.faifi.d@gmail.com (D.A.A.); zarbah.70777@gmail.com (A.M.Z.); sa9550088@gmail.com (S.A.A.); 2 Community Medicine Department, Faculty of Medicine, King Abdelaziz University, Jeddah 21589, Saudi Arabia; me@aaorg.org; 3 Clinical Pathology, University of Gezira, Wad Madani 5118, Sudan; moh.gasim6767@gmail.com (M.G.A.); frashad@moh.gov.sa (F.A.M.); 4 Microbiology Department, Khamis Mushait General Hospital, Khamis Mushait 62441, Saudi Arabia; maaabdelsalam@moh.gov.sa; 5 Public Health Department, Khamis Mushait General Hospital, Khamis Mushait 62441, Saudi Arabia; ss.albakri99@gmail.com; 6 Family and Community Medicine Department, College of Medicine, King Khalid University, Abha 61421, Saudi Arabia

**Keywords:** malaria, *Plasmodium vivax*, *Plasmodium falciparum*, imported malaria, severe malaria, outbreak, Saudi Arabia

## Abstract

Background: Saudi Arabia has made significant progress toward malaria elimination; however, imported cases continue to occur, particularly in the southwestern regions. This study aimed to describe the clinical characteristics and outcomes of patients with malaria in the Aseer Region, Saudi Arabia. Methods: A retrospective observational study was conducted at Khamis Mushait General Hospital, Aseer Region, Saudi Arabia, including all patients with malaria from January 2022 to December 2025. Demographic, clinical, laboratory, and outcome data were extracted from the electronic medical records. Severe malaria was defined according to the World Health Organization criteria. Multivariate logistic regression using Firth’s penalized maximum likelihood estimation was performed to identify independent predictors of severe malaria (≥1 WHO criterion). Statistical analysis was performed using R software (version 4.2.1). Results: A total of 311 patients were included, predominantly male (90.0%), with a mean age of 28.8 ± 11.3 years. Ethiopian nationals comprised nearly half the cases (48.2%), followed by Saudi (16.4%) and Yemeni (15.1%) nationals. *Plasmodium vivax* was the most common species (51.1%), followed by *Plasmodium. falciparum* (40.2%). Fever was the most frequent symptom (89.4%), followed by fatigue (50.8%), chills (46.9%), and vomiting (39.5%). Low parasitemia (<1%) was the most frequent finding (33.8%), followed by moderate (27.3%) and mild (18.3%) levels, while high (4.2%) and very high parasitemia (1.9%) were uncommon. Severe malaria (≥1 criterion) was diagnosed at 43.7%, with severe anemia (26.0%) and jaundice (23.2%) being the most frequent WHO severity criteria. Notably, 84% of the cases occurred during 2024–2025, indicating a recent outbreak, with a sharp peak of 43 cases in October 2024. Multivariate logistic regression identified two independent predictors of having at least one WHO severity criterion: higher parasitemia level (adjusted OR = 1.70 per 1% increase, 95% CI: 1.40–2.11, *p* < 0.001) and non-Saudi nationality (adjusted OR = 2.40, 95% CI: 1.10–5.62, *p* = 0.027). Conclusions: Malaria in the Aseer Region predominantly affects young adult male expatriates, suggesting its imported nature. The predominance of *P. vivax* represents a shift from historical patterns. Parasitemia level and being of non-Saudi nationality independently predict severe malaria and may therefore support risk stratification and clinical decision-making. The dramatic case surge in 2024–2025 highlights regional vulnerability to outbreaks despite control progress. These findings support enhanced screening for at-risk populations, maintenance of clinical capacity for severe malaria management, and robust surveillance systems for early outbreak detection.

## 1. Introduction

Malaria remains a major threat to global health. It is caused by multiple *Plasmodium (P)* species—*P. falciparum*, *P. vivax*, *P. malariae*, *P. ovale*, and *P. knowlesi* [1]. In 2024, an estimated 282 million malaria cases were reported in 80 endemic countries, representing an increase of around 9 million cases (3%) compared with 2023. More than half of this increase (58%) was reported in Ethiopia (+2.9 million cases), Madagascar (+1.9 million), and Yemen (+378,000) [2].

Malaria presents with a broad spectrum of clinical manifestations, ranging from mild illness to severe, life-threatening disease. Disease severity depends on the *Plasmodium* species, as well as the timing of diagnosis and initiation of treatment [3,4]. Severe malaria is characterized by organ dysfunction and hematological or metabolic abnormalities [4]. Complications include cerebral malaria, which can lead to long term neurological deficits, particularly in children, and severe anemia, especially among young children. In pregnant women, *P. falciparum* infection increases the risk of severe maternal illness, premature delivery, and low birth weight. Less common complications include splenic rupture, often associated with *P. vivax*, and nephrotic syndrome, which has been linked to *P. malariae* infection [5,6,7].

In endemic countries, despite the ongoing control efforts, malaria remains difficult to manage due to fragile healthcare systems, inadequate access to reliable diagnostic tools, and the high cost of preventive strategies, including insecticide-treated bed nets and antimalarial drugs [8]. Moreover, malaria poses a significant threat to both national and global economic development. Increased malaria transmission results in slower annual economic growth of 0.7% to 3% [9].

In the World Health Organization (WHO) Eastern Mediterranean Region, malaria cases declined from 6.9 million in 2000 to 4.3 million in 2015; however, this progress has recently reversed, with cases rising to 10.2 million in 2023. Countries like Pakistan and Afghanistan have experienced a recent surge in malaria incidence, partly linked to environmental factors, such as flooding [1]. This resurgence may also be influenced by the region’s diverse vector ecology, with a systematic review (1900–2024) identifying 45 Anopheles species across the region [10].

In the Arabian Peninsula, Yemen reported over 100,000 malaria cases in 2018, most of which were caused by *P. falciparum*. In the same year, Saudi Arabia recorded approximately 2700 confirmed cases, the majority of which were imported from countries such as Yemen, Pakistan, India, and Ethiopia [11,12]. Malaria control programs in Saudi Arabia have been highly effective, resulting in the interruption of local transmission since the 1940s. These measures entail indoor spraying, larviciding, case management, drug resistance monitoring, and community education, with a robust surveillance system enabling a rapid outbreak response [11,13,14]. Despite this progress, malaria cases are mainly diagnosed in the southwestern regions, particularly in Jazan and Aseer. In these areas, *P. falciparum* is the dominant species in the southwest, while *P. vivax* is more commonly reported in the northwest [15,16].

Although the measures have achieved a decline in malaria in Saudi Arabia, data on clinical presentation and outcomes in the southern region remain limited. A study conducted at Aseer Central Hospital (1991–1995), reported 334 confirmed malaria cases: 90.4% *P. falciparum* and 9.6% *P. vivax*. Among Saudis, 97.2% were diagnosed with *P. falciparum*, whereas expatriates were diagnosed with *P. vivax* (46.2%). Complications occurred in 13.8% of *P. falciparum* cases, including hemolysis requiring transfusion, cerebral malaria, and blackwater fever, with 0.7% mortality [17]. Between 2000 to 2015, locally acquired malaria in Aseer fell from 511 cases in 2000 to fewer than 40 cases annually during 2008–2014, rising slightly to 51 in 2015; all local infections were with *P. falciparum* [10]. According to WHO reports, 61 and 38 locally transmitted cases were recorded in 2018 and 2019, respectively [18,19]. Therefore, this study aims to describe the clinical characteristics, complications, and outcomes of malaria patients in the Aseer Region from 2022 to 2025, to inform clinical management, and to guide public health strategies.

## 2. Materials and Methods

### 2.1. Study Design and Setting

This retrospective observational study was conducted at Khamis Mushait General Hospital, Aseer Region of Saudi Arabia. This hospital serves as a major referral center in the region and provides care for both Saudi citizens and expatriate residents, with an estimated catchment population of approximately 600,000 individuals. The study period spanned January 2022 to December 2025.

### 2.2. Study Population

This study included patients with confirmed malaria infection diagnosed by microscopy or rapid diagnostic tests (RDTs) and were admitted or treated at the hospital during the study period. Patients with incomplete medical records or lacking confirmatory laboratory results were excluded. In total, 311 patients were included in the analysis.

### 2.3. Data Collection

Data were extracted from electronic medical records using a standardized data extraction tool. The following variables were collected:

- Demographic characteristics: age, sex, and nationality.
- Clinical presentation: documented symptoms at admission, including fever, fatigue, chills, vomiting, abdominal pain, headache, and neurological signs.
- Malaria species: *P. vivax*, *P. falciparum*, *P. ovale*, *P. malaria,* mixed infections, or unspecified species.
- Laboratory findings: hemoglobin levels, platelet counts, parasitemia levels, and blood culture results.
- Treatment regimen: Patients were managed as inpatients (for complicated cases) or outpatients (for uncomplicated cases), use of oral or intravenous antimalarial therapy, blood transfusions, renal replacement therapy, and intensive care unit (ICU) admission.
- In-hospital complications: based on the WHO severe malaria criteria, including severe anemia, jaundice, shock, renal impairment, cerebral malaria, pulmonary edema/acute respiratory distress syndrome, hypoglycemia, significant bleeding, and convulsions [20,21].
- Clinical outcomes: ICU admission and mortality.

### 2.4. Case Definitions

- Severe malaria: severe malaria was diagnosed based on the WHO criteria, including severe anemia (Hb < 7 g/dL), hyperparasitemia (>5%), cerebral malaria (Glasgow Coma Scale < 11), shock (systolic blood pressure < 80 mmHg), jaundice (bilirubin > 3 mg/dL), renal impairment (creatinine > 3 mg/dL), metabolic acidosis, pulmonary edema/acute respiratory distress syndrome, hypoglycemia, and significant bleeding [20].
- Anemia was defined and classified according to hemoglobin levels as mild (10–11.9 g/dL), moderate (7–9.9 g/dL), or severe (<7 g/dL).
- Thrombocytopenia was defined based on platelet count as mild (100–149 × 10^3^/µL), moderate (50–99 × 10^3^/µL), or severe (<50 × 10^3^/µL), following standard laboratory reference thresholds.
- Parasitemia levels: stratified as low (≤1%), moderate (2–5%), high (6–10%), and very high (>10%).

### 2.5. Statistical Analysis

Data analyses were performed using R (version 4.2.1). Descriptive statistics summarized the demographic, clinical, and laboratory characteristics of the study population. Categorical variables were presented as frequencies and percentages (*n*, %). Continuous variables were assessed for normality and are presented as range (minimum–maximum) and median with interquartile range (IQR). For comparative analyses, patients were stratified based on disease severity using the WHO criteria. Differences between the groups were evaluated using the Chi-squared test (or Fisher’s exact test when expected cell counts were less than 5), which was used to compare categorical variables such as sex, the prevalence of specific symptoms, complications, ICU admission, and mortality. For continuous variables (e.g., age, hemoglobin level, platelet count, parasitemia), the Kruskal–Wallis test was used for comparisons across three variables. A multivariate logistic regression analysis was performed to identify independent predictors of having at least one WHO severe malaria criterion. To assess the robustness of the findings, two models were constructed. Model A used the standard WHO definition of severe malaria, which includes hyperparasitemia (>5%) as a severity criterion. Model B applied an alternative definition excluding hyperparasitemia to avoid potential circularity, as parasitemia was included as a predictor of interest. Both models included the same a priori selected covariates based on clinical relevance and univariate screening (*p* < 0.10): age (continuous, years), sex, malaria species (*P. vivax* and *P. falciparum*), parasitemia level, and nationality (non-Saudi vs. Saudi). Due to sparse data and complete separation in some categories, Firth’s penalized maximum likelihood logistic regression was applied using the logistf package in R, providing bias-reduced and more stable parameter estimates, particularly in datasets with rare outcomes. Model performance was evaluated using the likelihood ratio test comparing each model to the null model, with statistical significance indicating improved fit over chance. McFadden’s pseudo-R^2^ was used as an indicator of model fit, and the Akaike information criterion (AIC) was used for model comparison. Results are reported as adjusted odds ratios (aOR) with 95% confidence intervals. A *p*-value of less than 0.05 was considered statistically significant for all tests.

### 2.6. Ethical Considerations

This study was conducted in accordance with the Declaration of Helsinki and approved by the Institutional Review Board (IRB) of Khamis Mushait General Hospital (REC-9-9-2024). As this study was conducted retrospectively, patient consent was waived. All data were anonymized prior to analysis to maintain patient confidentiality. We excluded incomplete and inconsistent data. All patients, whether treated as outpatients or discharged after hospitalization, were contacted for follow-up and advised returning if they experienced similar symptoms.

## 3. Results

A total of 311 patients were included, after excluding 9 participants with incomplete data. The majority were male (90.0%), with a median age of 26.0 [21.0, 33.0] years. Most participants were young adults aged 19–30 years (56.9%), followed by adults aged 31–45 years (22.2%). Children (12–18 years) accounted for 12.9% of cases, while middle-aged (46–60 years) and elderly (>60 years) individuals represented smaller proportions (6.1% and 1.9%, respectively). Patients were predominantly from Ethiopia (48.2%), Saudi Arabia (16.4%), and Yemen (15.1%), with smaller proportions from Sudan, Bangladesh, Pakistan, India, and other countries (Table 1).

**Figure 1:**
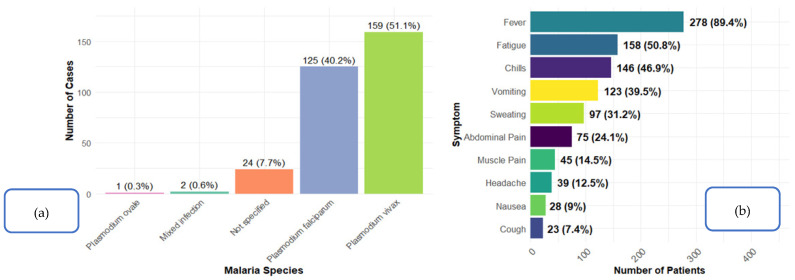
Distribution of malaria species and clinical symptoms among the study participants in the Aseer Region, Saudi Arabia, 2022–2025 (*n* = 311): (**a**) shows the distribution of malaria species, while (**b**) illustrates the distribution of clinical symptoms.

*P. vivax* was the most frequently identified species, accounting for 51.1% (*n* = 159) of infections, followed by *P. falciparum* (40.2%, *n* = 125). *P. ovale* was rare (0.3%, *n* = 1), and mixed infections were uncommon (0.6%, *n* = 2). A notable proportion of cases (7.7%, *n* = 24) were not specified (Figure 1a). Among the reported clinical manifestations, fever was the most common symptom, occurring in 89.4% (*n* = 278) of patients. This was followed by fatigue in 50.8% (*n* = 158), chills in 46.9% (*n* = 146), and vomiting in 39.5% (*n* = 123) of patients. Sweating was documented in 31.2% (*n* = 97) of patients, while abdominal pain was present in 24.1% (*n* = 75). Less frequent symptoms included myalgia (14.5%, *n* = 45), headache (12.5%, *n* = 39), nausea (9.0%, *n* = 28), and cough (7.4%, *n* = 23). Diarrhea was reported in 5.1% (*n* = 16) of patients, and convulsions were rare, observed in only 0.3% (*n* = 1) of cases (Figure 1b).

Anemia was highly prevalent, affecting 67.2% of patients. Normal hemoglobin levels were seen at only 32.8% of patients. Severe anemia (hemoglobin < 7 g/dL) was observed in 26.0% (*n* = 81) of patients, while moderate and mild anemia accounted for 21.5% (*n* = 67) and 19.6% (*n* = 61) of cases, respectively. The median hemoglobin level was 10.0 [6.6–13.0] g/dL. Thrombocytopenia was also common, with only 13.5% of patients demonstrating a normal platelet count. Moderate thrombocytopenia was the most frequent category (35.7%, *n* = 111), followed by severe (29.3%, *n* = 91) and mild thrombocytopenia (21.5%, *n* = 67). The median platelet count was 72.0 [45.0–118.0] × 10^3^/µL. Most patients had no documented complications (71.1%, *n* = 221), whereas 19.9% (*n* = 62) developed one complication and fewer than 10% experienced two or more complications. Blood transfusion was required in 28.6% (*n* = 89) of patients. Other complications included jaundice in 23.2% (*n* = 72) of patients, shock in 8.0% (*n* = 25), renal impairment in 3.9% (*n* = 12), cerebral malaria in 3.2% (*n* = 10), and renal replacement therapy in 1.3% (*n* = 4). Most patients (307, 98.7%) received artemisinin-based therapy as the WHO-recommended first-line treatment for malaria. Only a small proportion (*n* = 5, 1.6%) were treated with quinine-based therapy. A total of 290 patients (93.2%) required hospital admission and were managed as inpatients, of which 38 (12.2%) required ICU admission and 5 (1.6%) died (see Supplementary Table S1). Interestingly, rare complications were diagnosed as autoimmune hemolytic anemia (*n* = 4, 1.3%), hypocalcemia (*n* = 2, 0.6%), and one case developed splenic infarction (*n* = 1, 0.32%). (Table 2)

**Figure 2:**
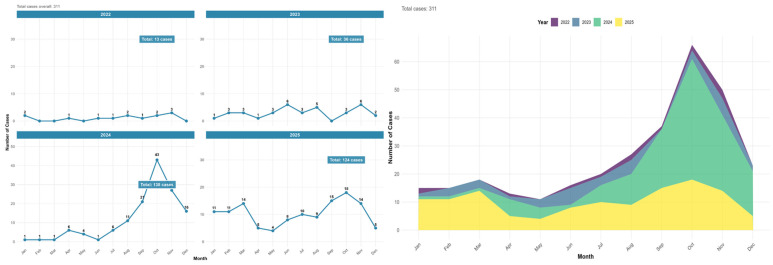
Temporal distribution of malaria cases in the Aseer Region, 2022–2025, showing a progressive increase with pronounced peaks in 2024 and sustained transmission into 2025.

Over a four-year study period, there was a shift from sporadic, low-level cases to a significant outbreak. In 2022 and 2023, the disease burden was minimal, with total cases of 13 and 36, respectively, and monthly counts rarely exceeded single digits. However, 2024 marks a critical turning point with a massive surge to 138 total cases, driven by a sharp epidemic peak in October, where 43 cases were recorded in a single month. This high level of transmission persisted into 2025, which recorded 124 total cases; although the peak magnitude was lower than the previous year (reaching 18 in October), the transmission season appeared more sustained, with notable activity in the spring and autumn (Figure 2).

**Figure 3:**
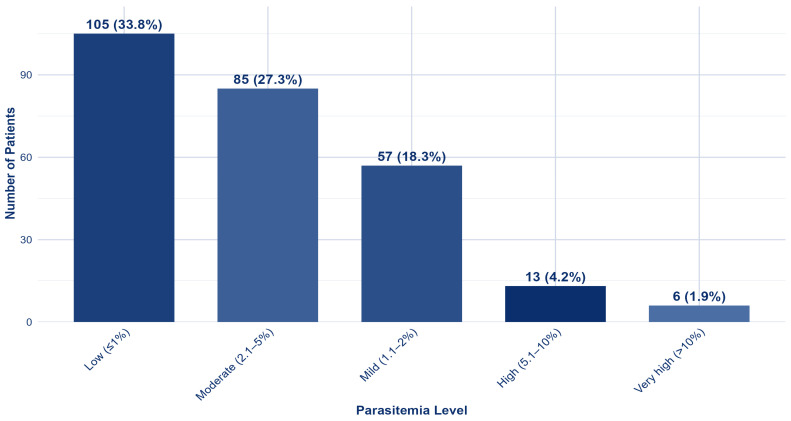
Distribution of parasitemia levels in hospitalized malaria patients, Aseer Region, 2022–2025 (*n* = 266).

The parasitemia distribution among the 311 malaria patients showed that most infections were of low to moderate intensity. Low parasitemia (<1%) was the most common, observed in 105 patients (33.8%), followed by moderate parasitemia (2.1–5%) in 85 patients (27.3%) and mild parasitemia (1.1–2%) in 57 patients (18.3%). High parasitemia (5.1–10%) was seen in 13 patients (4.2%), and very high parasitemia (>10%) was rare, affecting only 6 patients (1.9%) (Figure 3). Among those with very high parasitemia, 17/19 (89.5%) were diagnosed with *P. falciparum* (Supplementary Figure S1).

Among the WHO severe malaria criteria, severe anemia (hemoglobin < 7 g/dL) was the most frequently observed manifestation, present in 26.0% (*n* = 81) of cases. Jaundice was the second most common criterion, occurring in 23.2% (*n* = 72) of cases, followed by shock in 8.0% (*n* = 25) and hyperparasitemia (>5%) in 6.1% (*n* = 19) of cases. Metabolic acidosis and renal impairment were each identified in 3.9% (*n* = 12) of patients, while cerebral malaria was documented in 3.2% (*n* = 10) of patients. Less frequent complications included pulmonary edema/acute respiratory distress syndrome (1.6%, *n* = 5), hypoglycemia (1.3%, *n* = 4), and significant bleeding (0.6%, *n* = 2) (Table 3).

More than half of the patients had no severity criteria (*n* = 175, 56.2%), followed by those with one criterion (*n* = 84, 27.0%) and those with two or more criteria or death (*n* = 52, 16.7%). Age was comparable across the groups (*p* = 0.537). Most patients were male, with no significant difference in sex distribution between the groups (*p* = 0.358). The distribution of malaria species did not differ significantly between the groups (*p* = 0.400). However, *P. falciparum* showed a relatively higher proportion among patients with ≥2 severity criteria or death (22.4% within species), whereas *P. vivax* was more frequently observed among patients without a severity criterion (55.3%) and those with a single criterion (30.8%). Hemoglobin levels declined markedly with increasing severity, from 11.0 g/dL in patients without a criterion to 8.0 g/dL in those with one criterion and 6.0 g/dL in those with ≥2 criteria or death (*p* < 0.001). A similar significant reduction was observed in platelet counts (82.0, 71.0, and 47.5 × 10^3^/µL, respectively; *p* < 0.001). By contrast, parasitemia increased with severity, reaching a median of 3.0% in the most severe group compared with 1.0% and 2.5% in the no-criteria and one-criterion groups (*p* < 0.001). Clinical outcomes showed a marked gradient, with ICU admission increasing from 10.5% in both the no-criteria and one-criterion groups to 78.9% in those with ≥2 criteria or death (*p* < 0.001). Mortality occurred exclusively in the most severe group (100% of deaths; *p* < 0.001) (Table 4). The comparison between *P. falciparum* and *P. vivax* is shown in Supplementary Table S3.

**Figure 4:**
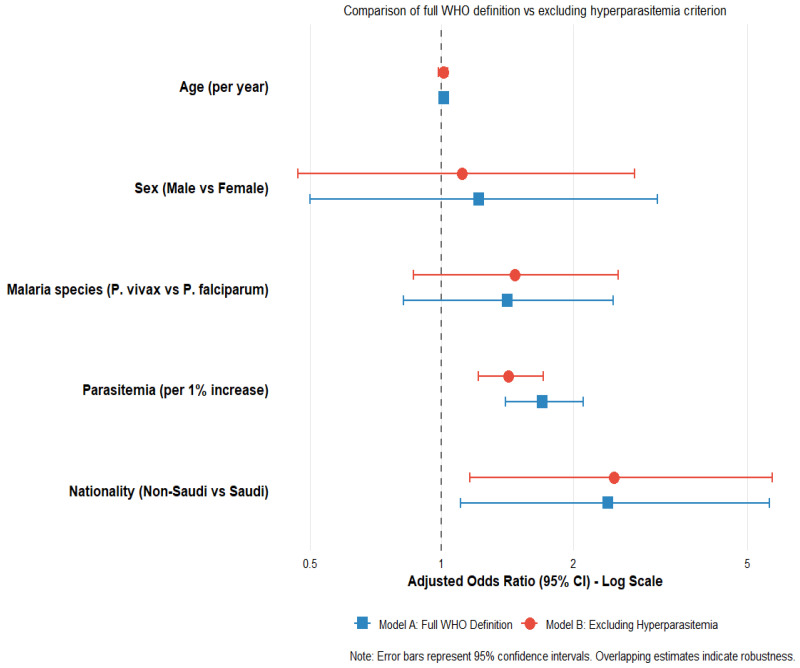
Forest Plot of Multivariate Logistic Regression Analysis for Factors Associated with ≥1 WHO Severity Criteria Among Malaria Patients, Aseer Region, Saudi Arabia (2022–2025).

The forest plot demonstrates that higher parasitemia (aOR = 1.70, 95% CI: 1.40–2.11, *p* < 0.001) and non-Saudi nationality (aOR = 2.40, 95% CI: 1.10–5.62, *p* = 0.027) were independently associated with increased odds of having ≥1 WHO severity criteria. The model was statistically significant (Likelihood Ratio χ^2^ = 50.34, df = 5, *p* < 0.001). See Figure 4 and Supplementary Tables S4 and S5.

The forest plot shows the adjusted odds ratios (aOR) with 95% confidence intervals from multivariate logistic regression analysis. The dashed vertical line at aOR = 1 indicates no association. Squares represent point estimates, and horizontal lines represent 95% confidence intervals. Significance levels are indicated as follows: *** *p* < 0.001, ** *p* < 0.01, * *p* < 0.05. The model was adjusted for age, sex, malaria species, parasitemia level, and nationality.

## 4. Discussion

Saudi Arabia has made significant progress toward malaria elimination; however, malaria has remained endemic in the southwestern regions of the country. The Government has made an outstanding political and financial commitment to a malaria elimination strategy since 2000 within the framework of the national plan for socioeconomic development of the southwestern regions [22]. This study highlights that hospitalized malaria in the Aseer region predominantly affected young adult males and was largely composed of probably imported cases, with Ethiopian nationals representing a substantial proportion of patients. *P. vivax* was the most frequently identified species, followed by *P. falciparum*. Clinical presentation was dominated by fever, while a considerable proportion of patients developed complications, particularly hematological and hepatic manifestations consistent with the WHO severity criteria. Thrombocytopenia was highly prevalent, with a notable subset of patients experiencing severe reductions. Overall, a large proportion of patients required ICU admission management, and mortality remained low. The findings also suggest a temporal concentration of cases in the most recent years of the study period, indicating a possible recent increase in transmission or importation. Importantly, severity was associated with worsening laboratory parameters, including declining hemoglobin and platelet counts and increasing parasitemia, reinforcing the established markers of disease severity in malaria. The multivariate logistic regression analysis identified higher parasitemia and being non-Saudi as the main independent predictors of having at least one WHO severity criterion.

The overwhelming predominance of young adult males and foreign nationals, particularly from Ethiopia and Yemen, confirms that malaria in this region remains primarily an imported disease. Imported malaria refers to cases in which the infection was acquired from outside the region, which is different from indigenous cases, where it is locally circulating without evidence of importation, and introduced malaria, which is caused by transmission from imported case [23]. This pattern aligns with previous Saudi Arabian studies showing that malaria cases are concentrated among expatriate workers from endemic countries [24,25,26]. The movement of individuals infected with malaria may result in the reintroduction of malaria in areas where malaria has been eliminated [27]; moreover, this contributes to the spread of drug-resistant parasite strains [28] and strained relations between neighboring countries that are employing differing control measures [29].

The median age of 26.0 years reflects the demographic profile of the labor force in the region. The high prevalence among young adult males reflects the demographic profile of the expatriate labor force in southern Saudi Arabia, where migrant workers—often employed in construction, agriculture, and manual labor—arrive from malaria-endemic countries. These expatriates likely acquired infections in their countries prior to arrival or during visits, rather than through local transmission. This finding emphasizes that, despite successful elimination efforts in most of Saudi Arabia, imported cases continue to establish a challenge, particularly in regions with large expatriate populations. The findings emphasize the importance of targeted screening and preventive measures for high-risk populations, particularly incoming expatriates from countries endemic with malaria.

The predominance of *P. vivax* (51%) over *P. falciparum* (40%) is noteworthy, as historical data from southwestern Saudi Arabia has typically shown *P. falciparum* dominance [22]. This shift may reflect changing patterns of imported cases, possibly related to increased population movement from *P. vivax*-endemic regions. However, *P. falciparum* was disproportionately associated with nearly all hyperparasitemic infections (90%), consistent with its well-established propensity for causing severe complications [30]. This finding underscores the need for continued attention for severe *P. falciparum* infection despite its relatively lower overall prevalence. Moreover, surveillance systems should be strengthened to detect and monitor imported malaria early, and may contribute to the change in epidemiological patterns.

The near-universal presence of fever aligns with classical malaria descriptions, though the relatively low frequency of headache and other classic symptoms may reflect incomplete documentation in retrospective records or atypical presentations in this population. The high prevalence of severe anemia and thrombocytopenia is consistent with previous studies from endemic areas. Thrombocytopenia is as a marker of malaria infection specifically if it is associated with fever in endemic area [31,32,33]. Thrombocytopenia is a commonly reported feature of *P. falciparum* and *P. vivax* malaria; however, it is not a sign of severity [34]. Severe anemia, the most frequent WHO severity criterion, likely results from a combination of hemolysis (parasitized and non-parasitized RBCS), bone marrow decreased production, possibly nutritional deficiencies, and pre-existing anemia in this predominantly expatriate population [21,35]. The substantial requirement for blood transfusion (29%) reflects the clinical significance of this complication. The 43.7% proportion of severe malaria cases is comparable to the rates reported from other endemic settings in Sub-Saharan Africa and South India [36,37]. Jaundice emerged as the second most common severe manifestation (23%), which may reflect both hemolysis and hepatocellular dysfunction, especially with *P. falciparum* [38]. By contrast, a lower incidence (10.1%) was observed among patients with *P. vivax* infection. Previous studies have reported jaundice in 32% of patients with normal liver enzymes, increasing to 66% among those with elevated liver enzymes [37,39]. The relatively low rates of cerebral malaria (3.2%) and renal impairment (3.9%) are encouraging and may reflect relatively early presentation and access to care, though the retrospective design may underestimate these complications if not consistently documented.

In this study, very uncommon complications were developed, namely autoimmune hemolytic anemia, hypocalcemia, and splenic infarction. Autoimmune hemolytic anemia may be due to infection-induced immune dysregulation with autoimmune antibodies against the membrane lipid phosphatidylserine leading to destruction of parasitized and non-parasitized erythrocytes [40]. The concern of autoimmune hemolytic anemia was considered when most patients responded to supportive care and transfusion, while a subset developed refractory anemia despite confirmed parasite clearance and repeated transfusions. In these cases, autoimmune hemolytic anemia was suspected, supported by positive direct antiglobulin (Coombs) tests in all affected patients. Moreover, the short time frame made delayed artesunate-associated hemolysis unlikely. Hypocalcemia is usually multifactorial; it may be caused by renal impairment, hypoalbuminaemia, inflammatory cytokine release, reduced nutritional intake or, in some cases, the effects of blood transfusion and transient metabolic alterations during severe illness. In this study, two cases had persistent hypocalcemia that was improved by calcium replacement. Testing for hypoparathyroidism revealed associated transient hypoparathyroidism that improved later. Similar cases of hypocalcemia were reported with *P. falciparum* infection [41]. Splenic infarction may be due to acute splenic enlargement with vascular congestion, microvascular obstruction parasitized erythrocytes, and the hypercoagulable state associated with malaria and anemic hypoxia, all of which compromise splenic blood flow and lead to focal ischemia. Together, these findings reflect the hematological, immunological, vascular, and metabolic disturbances that characterize severe malaria [42].

The dramatic surge in cases during 2024–2025, with 84% of all study cases concentrated in these two years and a sharp peak of cases in October 2024, represents a significant epidemiological event. The reduced number of cases during 2022–2023 is mainly due to the restriction of travel to limit the COVID-19 transmission. As we mentioned, most cases of malaria are imported, which explains the low number of reported cases in these two years. While, in 2024 and 2025, the increased pattern suggests either a large-scale importation of cases, possibly related to population movements from neighboring Yemen or the Horn of Africa, or a localized outbreak following introduction. The timing coincides with post-flooding conditions in several endemic countries to help establish a local reservoir [26].

The global malaria mortality rate for 2023 is estimated at 13.7 deaths per 100,000 individuals [43]. In this study, the overall mortality of 1.6% compares favorably with global estimates. A meta-analysis of severe imported malaria reported a pooled mortality of 5.1% among severe cases [44]; other estimates from endemic settings have ranged from 12% to 17% [45,46], suggesting adequate case management at this regional referral center. However, the high ICU admission rate (12%) and blood transfusion requirement (29%) indicate substantial healthcare resource utilization. The difference in case fatality rate may reflect differences in presentation timing or case mix during the outbreak. The fact that only 1.3% required renal replacement therapy suggests that acute kidney injury, when present, was generally manageable or less severe than in some other settings.

The strong associations between disease severity and lower hemoglobin, lower platelet counts, and higher parasitemia are physiologically reasonable and consistent with established pathophysiology [47,48]. The finding that hemoglobin declined progressively, from 11.0 g/dL in non-severe cases (no severity criteria) to 6.0 g/dL in severe cases, underscores the cumulative hematological insult in severe malaria. Similarly, the marked difference in parasitemia (1.0% vs. 3.0% in severe) reinforces parasite burden as a key determinant of clinical outcomes. The low presentation of female patients in the severity analysis with male predominance, limits conclusions about sex-based differences in disease progression.

In this study, 43.8% of patients presented with severe malaria. This substantial proportion may indicate delayed arrival at healthcare facilities, especially among expatriate groups, including those with irregular residency status who may encounter obstacles to accessing prompt medical care. Referral bias in hospital-based settings, where more serious cases are disproportionately represented, may also contribute. Furthermore, the inclusion of non-immune individuals may heighten vulnerability to severe disease. The main predictor of malaria severity was being non-Saudi. Likewise, a study by Malik et al. [17] reported that malaria showed a poorer response to chloroquine in expatriates compared with Saudis. This observation may indicate later presentation for care, reduced baseline immunity, or obstacles to accessing healthcare among expatriate populations. Parasitemia level was a significant predictor of malaria severity with each one percent increase in parasitemia associated with a 70% increase in the odds of severe malaria. The significant effect of parasitemia level remained even if the criteria of parasitemia of >5% was removed from the case definition of severe malaria. Moreover, *Plasmodium* density was found to be significantly associated with hematological impairment. This association may be attributed to enhanced red blood cell destruction, suppression of bone marrow activity, and immune-mediated processes that occur in the context of higher parasite burdens. In addition to parasitemia, transmission rates are important. A study in Thailand found that even low parasite density of 0.5% was associated with severe disease in areas with low transmission [49]. These results underscore that parasitemia should be viewed as a continuous risk parameter, rather than depending solely on fixed cut-off values when evaluating malaria severity.

### 4.1. Study Limitations

Several limitations warrant consideration. The retrospective design relying on medical records may underestimate complications and symptoms not systematically documented. The single-center setting may limit generalizability to other regions of Saudi Arabia. Parasitemia was not measured in 14.5% of patients, potentially biasing severity assessments. The absence of follow-up data precludes an evaluation of long-term outcomes, particularly for patients with severe anemia or cerebral malaria who may experience lasting sequelae. Additionally, the study period captured an outbreak situation, and the findings may not reflect inter-epidemic periods. Furthermore, the lack of systematically recorded travel history and time since arrival limited the ability to definitively distinguish imported from locally acquired infections.

### 4.2. Public Health Implications

These findings have several implications for malaria control in the region. First, enhanced screening and preventive measures for incoming expatriates from endemic countries, particularly Ethiopia and Yemen, could reduce imported cases. Second, healthcare facilities should maintain readiness for malaria outbreaks, ensuring adequate supplies of blood products, antimalarial medications, and ICU capacity. Third, the predominance of *P. vivax* warrants attention to radical cure protocols to prevent relapses. Fourth, the outbreak pattern observed in 2024–2025 underscores the need for robust surveillance systems capable of early outbreak detection and rapid response. Finally, because this study found a strong association between rising parasitemia and disease severity, clinical practice should prioritize early diagnosis and the careful tracking of parasite levels to help prevent the development of severe malaria.

## 5. Conclusions

This study demonstrates that malaria in the Aseer region predominantly affects young adult male expatriates, with *P. vivax* now more common than *P. falciparum*, though the latter remains responsible for most severe disease. Severe complications occur in 28.9% of patients, with severe anemia and jaundice being most frequent. Non-Saudi nationality and the degree of parasitemia emerged as independent predictors of severe malaria. Importantly, this relationship remained significant even when hyperparasitemia (>5%) was removed from the severity criteria, highlighting the standalone prognostic importance of parasitemia considered as a continuous risk factor. The dramatic case surge in 2024–2025 highlights the region’s vulnerability to malaria outbreaks despite overall control progress. These findings highlight the importance of enhancing surveillance systems, implementing focused prevention efforts for high-risk groups, and maintaining robust clinical readiness to manage severe malaria effectively. Dedicated screening initiatives for expatriate communities, especially recent arrivals and individuals with limited access to medical services—should be prioritized. Furthermore, strengthening vector control interventions, such as indoor residual spraying and larval source management in areas with elevated risk, is essential. Initiatives to engage communities, to increase awareness, to encourage prompt healthcare-seeking behavior, and to promote adherence to preventive practices should also be implemented.

## Figures and Tables

**Table 1:** Demographic Characteristics of Hospitalized Malaria Patients, Aseer Region, 2022–2025 (*n* = 311).

Variables	Level	*n* = 311
Sex	Female	31 (10.0%)
	Male	280 (90.0%)
Age	Median (Q1, Q3)	26.0 [21.0, 33.0]
	Range	12.0–94.0
	Children (12–18)	40 (12.9%)
	Young adults (19–30)	177 (56.9%)
	Adults (31–45)	69 (22.2%)
	Middle-aged (46–60)	19 (6.1%)
	Elderly (>60)	6 (1.9%)
Nationality	Ethiopian	150 (48.2%)
	Saudi Arabian	51 (16.4%)
	Yemeni	47 (15.1%)
	Sudanese	20 (6.4%)
	Bangladeshi	11 (3.5%)
	Pakistani	9 (2.9%)
	Indian	8 (2.6%)
	Egyptian	4 (1.3%)
	Somali	2 (0.6%)
	Ugandan	2 (0.6%)
	Afghani	1 (0.3%)
	Ghanaian	1 (0.3%)
	Other	5 (1.6%)

Data are presented as number (percentage). Age is presented as median with interquartile range (Q1–Q3).

**Table 2:** Laboratory Parameters, Complications, and Clinical Outcomes Among Malaria Patients, Aseer Region, 2022–2025 (*n* = 311).

Characteristic		*n* = 311
Hemoglobin (g/dL)	Median [Q1–Q3]	10.0 [6.6–13.0]
	Range	2.1–17.6
Anemia status	Normal (≥12 g/dL)	102 (32.8%)
	Mild (10–11.9 g/dL)	61 (19.6%)
	Moderate (7–9.9 g/dL)	67 (21.5%)
	Severe (<7 g/dL)	81 (26.0%)
Platelet count (×10^3^/µL)	Median [Q1–Q3]	72.0 [45.0–118.0]
	Range	3.0–441.0
Thrombocytopenia status	Normal (≥150 × 10^3^/µL)	42 (13.5%)
	Mild (100–149 × 10^3^/µL)	67 (21.5%)
	Moderate (50–99 × 10^3^/µL)	111 (35.7%)
	Severe (<50 × 10^3^/µL)	91 (29.3%)
Number of complications	0	221 (71.1%)
	1	62 (19.9%)
	2	17 (5.5%)
	3	5 (1.6%)
	4	2 (0.6%)
	5	1 (0.3%)
	6	3 (1.0%)
Complications	Severe anemia requiring blood transfusion	89 (28.6%)
	Jaundice	72 (23.2%)
	Shock	25 (8.0%)
	Renal impairment	12 (3.9%)
	Cerebral malaria	10 (3.2%)
	Renal replacement therapy	4 (1.3%)
	Autoimmune hemolytic anemia	4 (1.3%)
	Hypocalcemia	2 (0.6%)
	Splenic infarction	1 (0.3%)
Antimalaria therapy	Artemisinin-based therapy	307 (98.7%)
	Quinine-based therapy	4 (1.3%)
Hospital admission (Yes)		290 (93.2%)
Intensive care unit admission		38 (12.2%)
Death (Yes)		5 (1.6%)

Data are presented as *n* (%) for categorical variables and median with interquartile range [Q1–Q3] for non-normally distributed variables. All patients with *P. vivax* were treated with hypnozoitocidal therapy to eliminate dormant hypnozoites and to prevent recurrence. For details on treatment refer to Supplementary Table S1.

**Table 3:** Distribution of the WHO severe malaria criteria, Aseer Region, 2022–2025 (*n* = 311).

Criterion	Cases	Percentage
Severe anemia (Hb < 7 g/dL)	81 (26.0%)	
Jaundice (bilirubin > 3 mg or >50 µmol/L)	72 (23.2%)	
Shock (systolic blood pressure < 80 mmHg)	25 (8.0%)	
Hyperparasitemia (>5%)	19 (6.1%)	
Metabolic acidosis (HCO^3^ < 15 or Lactate ≥ 5)	12 (3.9%)	
Renal impairment (Creatinine > 3 mg/dL or >265 µmol/L or Urea > 20)	12 (3.9%)	
Cerebral malaria (Glasgow coma scale < 11)	10 (3.2%)	
Pulmonary edema/acute respiratory distress syndrome	5 (1.6%)	
Hypoglycemia (Glucose < 2.2 mmol/L or <40 mg)	4 (1.3%)	
Significant bleeding	2 (0.6%)	
Multiple convulsions	0 (0.0%)	

Severe malaria was defined as the presence of one or more World Health Organization (WHO) severity criteria. Hyperparasitemia was defined as >5% parasitized red blood cells. Hypoglycemia was defined as blood glucose < 2.2 mmol/L (<40 mg/dL). Metabolic acidosis was defined as bicarbonate < 15 mmol/L or lactate ≥ 5 mmol/L. Renal impairment was defined as creatinine > 3 mg/dL or urea > 20 mmol/L.

**Table 4:** Clinical and laboratory characteristics across the WHO severity categories among malaria patients (*n* = 311).

Characteristics	No Criteria *n* = 175 (56.2%)	1 Criterion *n* = 84 (27.0%)	≥2 Criteria or Death *n* = 52 (16.7%)	Test Statistics	*p*-Value
**Age (years)**	26.0 [21.0–33.0]	25.0 [21.0–32.0]	26.0 [21.5–32.0]	H = 1.24	0.537
**Sex**				χ^2^ = 2.07	0.358
Male	155 (55.4%)	79 (28.2%)	46 (16.4%)		
Female	20 (64.5%)	5 (16.1%)	6 (19.4%)		
**Malaria species**				χ^2^ = 8.67	0.4
* Plasmodium falciparum*	68 (54.4%)	29 (23.2%)	28 (22.4%)		
* Plasmodium ovale*	1 (100%)	0 (0.0%)	0 (0.0%)		
* Plasmodium vivax*	88 (55.3%)	49 (30.8%)	22 (13.8%)		
Mixed infection	2 (100%)	0 (0%)	0 (0%)		
Not specified	16 (66.7%)	6 (25.0%)	2 (8.3%)		
**Hemoglobin (g/dL)**	11.0 [9.4–13.5]	8.0 [5.5–11.9]	6.0 [4.2–7.6]	H = 91.79	<0.001
**Platelets (×10^3^/µL)**	82.0 [53.0–134.0]	71.0 [45.5–111.5]	47.5 [31.0–94.0]	H = 16.10	<0.001
**Parasitemia (%)**	1.0 [1.0–2.0]	2.5 [2.0–3.0]	3.0 [2.0–10.0]	H = 47.45	<0.001
**ICU admission**	4 (10.5%)	4 (10.5%)	30 (78.9%)	χ^2^ = 120.71	<0.001
**Mortality**				χ^2^ = 25.31	<0.001
Died	0 (0.0%)	0 (0.0)	5 (100%)		
Survived	175 (57.2%)	84 (27.5%)	47 (15.4%)		

Data are presented as *n* (%) or median (interquartile range). Percentages are calculated within each severity group. ICU: intensive care unit. For continuous variables (Age, Hemoglobin, Platelets, Parasitemia), the Kruskal–Wallis H test was used. This non-parametric test was chosen because the data were not normally distributed (Shapiro–Wilk test, *p* < 0.05) and the groups had unequal variances (Levene’s test, *p* < 0.05). The Kruskal–Wallis test compares medians across the three groups without assuming normal distribution. For categorical variables (Sex, Malaria species, ICU admission, Mortality), the Chi-squared test (χ^2^) was used. Fisher’s exact test with a simulated *p*-value was applied when any expected cell count was <5 to ensure an accurate *p*-value calculation. Significant findings (*p* < 0.05).

## Data Availability

Data used in this analysis are available as Supplementary Material Table S2.

## Supplementary Materials

The following supporting information can be downloaded at: https://www.mdpi.com/article/10.3390/tropicalmed11040108/s1, Figure S1: Prevalence and Species Distribution of Hyperparasitemia Among Malaria Patient; Supplementary Table S1: Distribution of Antimalarial Treatment Regimens Among Patients, Aseer Region, 2022–2025 (N = 311); Supplementary Table S2: Comparison of Hospitalized vs. Outpatient Malaria Patients; Supplementary Table S3: Comparison of *Plasmodium falciparum* vs. *Plasmodium vivax* Malaria; Supplementary Table S4: Univariate and Multivariate Logistic Regression Analysis for Factors Associated with ≥1 WHO Severity Criteria; Supplementary Table S5: Multivariate Logistic Regression for ≥1 WHO Severity Criteria (Sensitivity Analysis).
