# Supplementary material for: Clinical Characteristics and Outcomes of Malaria Patients in the Aseer Region, Saudi Arabia: A Retrospective Study (2022–2025)
Source: Trop Med Infect Dis. 2026 Apr 20;11(4):108. doi: 10.3390/tropicalmed11040108 (PMC13120656; doi:10.3390/tropicalmed11040108)
Supplement: Supplementary file 1 [file tropicalmed-11-00108-s001.zip › tropicalmed-4204366-supplementary.pdf]

Supplementary tables

Most patients were treated with intravenous artesunate (268; 86.2%), making it the most frequently used antimalarial therapy. Artesunate combined with sulfadoxine–pyrimethamine was administered in 39 cases (12.5%), while quinine plus clindamycin/Doxycycline was used in a small proportion of patients (4; 1.3%). **Table S1**

Supplementary Table S1: Distribution of Antimalarial Treatment Regimens Among Patients, Aseer Region, 2022–2025 (N = 311)

Medication	N (%)
Artesunate plus sulfadoxine–pyrimethamine	39 (12.5)*
Intravenous Artesunate (I.V)	268 (86.2)
Quinine plus clindamycin (I.V)	2(0.6)
Quinine plus Doxycycline (I.V)	2(0.6)

Of all patients on intravenous (I.V) medication, 73 shifted to oral medication (Artesunate Plus Doxycycline or atovaquone-proguanil),* oral medication was stated for 9 cases with severe malaria due to unavailability of IV medication

Hospitalized patients had significantly lower hemoglobin levels (median 10.0 g/dL vs 14.0 g/dL, $p < 0.001$) and lower platelet counts (median 70.5 vs 117.7 $\times 10^3/\mu L$, $p = 0.013$) compared to outpatients, indicating more severe hematological abnormalities requiring admission. The proportion of patients meeting WHO severe malaria criteria was dramatically higher among hospitalized patients (50% vs 7.7%, $p = 0.003$). Additionally, 13% of hospitalized patients required ICU admission, while no outpatients needed intensive care. Age and sex distribution were similar between both groups, with no significant differences ($p = 0.209$ for age, $p = 0.451$ for sex). Notably, Ethiopian nationals constituted 51% of hospitalized patients but only 19% of outpatients, while Saudis represented 16% of hospitalized and 24% of outpatients. However, these nationality differences did not reach statistical significance. Malaria species distribution was similar between groups, with P. vivax being the most common species in both (52% hospitalized, 38% outpatient), followed by P. falciparum (40% hospitalized, 38% outpatient). Parasitemia levels showed a trend toward higher values in hospitalized patients (median 2.0% vs 1.0%), though this difference was not statistically significant ($p = 0.08$). Mortality was very low overall, with five deaths (1.7%) occurring exclusively in the hospitalized group. **Supplementary Table S2**

Supplementary Table S2: Comparison of Hospitalized vs Outpatient Malaria Patients

Characteristic	Hospitalized (N = 290)	Outpatient (N = 21)	p–value
**Age (years)**	26.0 [21.0–32.0]	30.0 [22.0–37.0]	0.209
**Sex**			0.451
Female	28 (9.7%)	3 (14%)	

Male	262 (90%)	18 (86%)	
**Nationality**			
Bangladeshi	8 (2.8%)	3 (14%)	
Egyptian	5 (1.7%)	0 (0%)	
Ethiopian	148 (51%)	4 (19%)	
Ghanaian	1 (0.3%)	0 (0%)	
Indian	8 (2.8%)	0 (0%)	
Pakistani	6 (2.1%)	3 (14%)	
Saudi Arabian	45 (16%)	5 (24%)	
Somali	2 (0.7%)	0 (0%)	
Sudanese	14 (4.8%)	5 (24%)	
Ugandan	2 (0.7%)	0 (0%)	
Unknown/Not specified	4 (1.4%)	0 (0%)	
Yemeni	47 (16%)	1 (4.8%)	
**Malaria species**			0.094
Mixed infection	2 (0.7%)	0 (0%)	
Not specified	19 (6.6%)	5 (24%)	
Plasmodium falciparum	117 (40%)	8 (38%)	
Plasmodium ovale	1 (0.3%)	0 (0%)	
Plasmodium vivax	151 (52%)	8 (38%)	
**Hemoglobin (g/dL)**	10.0 [6.7–13.0]	14.0 [10.6–15.0]	<0.001
**Platelets ($\times 10^3/\mu L$)**	70.5 [45.0–116.0]	117.7 [74.0–134.0]	0.013
**Parasitemia (%)**	2.0 [1.0–3.0]	1.0 [1.0–2.5]	0.08
**Severe malaria (WHO criteria)**	135 (50%)	1 (7.7%)*	0.003
**Intensive care unit admission**	38 (13%)	0 (0%)	0.089
**Mortality**			0.999
Died	5 (1.7%)	0 (0%)	
Survived	285 (98%)	21 (100%)	

Case of severe malaria refused to be admitted the severity criteria was Jaundice (Bilirubin>3 mg or >50 μmol/L).

Comparison between patients infected with P.falciparum (n=125) and P.vivax (n=159) revealed that age distribution was nearly identical, with a median age of 26 years for P. falciparum patients and 25 years for P. vivax patients (p = 0.954). Both groups were predominantly male, with 92% of P. falciparum and 89% of P. vivax patients being male. Nationality distribution showed some variation, with Ethiopian nationals being more common among P. vivax cases (57% vs 45%),

while Saudi and Yemeni nationals were more frequent among P. falciparum cases (20% and 21% vs 13% and 13%, respectively). However, these differences were not statistically significant. Laboratory parameters showed no significant differences between species. Hemoglobin levels were comparable between P. falciparum (median 9.8 g/dL) and P. vivax (median 10.0 g/dL) patients (p = 0.923). Platelet counts were also similar, with medians of 76.0 and 69.0 $\times 10^3/\mu L$, respectively (p = 0.872). Parasitemia levels showed no statistically significant difference, though P. falciparum had a slightly higher median (2.0% vs 2.0%, p = 0.189) and a wider upper range (4.0% vs 3.0%). Clinically, P. falciparum was associated with more severe neurological and renal complications. Cerebral malaria occurred significantly more frequently in P. falciparum patients (6.4% vs 0.6%, p = 0.012). Renal impairment was observed exclusively in P. falciparum patients (9.6% vs 0%, p < 0.001). Jaundice was more common in P. falciparum cases (27% vs 21%), though this difference did not reach statistical significance (p = 0.209). Shock showed a similar pattern (11% vs 6.9%, p = 0.214). Despite these differences in specific complications, overall severe malaria rates were nearly identical. Severe malaria by WHO criteria was present in 48% of P. falciparum patients and 47% of P. vivax patients (p = 0.999). ICU admission rates were higher in P. falciparum patients (16% vs 10%), though not statistically significant (p = 0.153). Mortality was low in both groups, with four deaths (3.2%) among P. falciparum patients and one death (0.6%) among P. vivax patients, a difference that did not reach statistical significance (p = 0.173). Hospitalization rates were nearly identical (94% vs 95%). **Supplementary Table S3**

Supplementary Table S3: Comparison of *Plasmodium falciparum vs Plasmodium vivax* Malaria

**Characteristic**	**Plasmodium falciparum**<br><br>**N = 125**	**Plasmodium vivax**<br><br>**N = 159**	**p–value2**
**Age (years)**	26.0 [20.0–33.0]	25.0 [22.0–33.0]	0.954
**Sex**			0.425
Female	10 (8.0%)	18 (11%)	
Male	115 (92%)	141 (89%)	
**Nationality**			
Bangladeshi	4 (3.2%)	4 (2.5%)	
Egyptian	0 (0%)	1 (0.6%)	
Ethiopian	56 (45%)	90 (57%)	
Indian	2 (1.6%)	5 (3.1%)	
Pakistani	2 (1.6%)	7 (4.4%)	
Saudi Arabian	25 (20%)	21 (13%)	
Somali	0 (0%)	2 (1.3%)	
Sudanese	5 (4.0%)	8 (5.0%)	

Ugandan	2 (1.6%)	0 (0%)	
Unknown/Not specified	3 (2.4%)	0 (0%)	
Yemeni	26 (21%)	21 (13%)	
**Hemoglobin (g/dL)**	9.8 [7.0–12.0]	10.0 (6.3–13.0]	0.923
**Platelets (×10³/μL)**	76.0 [41.0–125.0]	69.0 (47.0–114.0]	0.872
**Parasitemia (%)**	2.0 [1.0–4.0)	2.0 [1.0–3.0]	0.189
**ICU admission**	20 (16%)	16 (10%)	0.153
**Cerebral malaria**	8 (6.4%)	1 (0.6%)	0.012
**Renal impairment**	12 (9.6%)	0 (0%)	<0.001
**Jaundice**	34 (27%)	33 (21%)	0.209
**Shock**	14 (11%)	11 (6.9%)	0.214
**Severe malaria (WHO criteria)**	57 (48%)	71 (47%)	0.999
**Mortality**			0.173
Died	4 (3.2%)	1 (0.6%)	
Survived	121 (97%)	158 (99%)	
**Hospitalization status**			0.617
Hospitalized	117 (94%)	151 (95%)	
Outpatient	8 (6.4%)	8 (5.0%)	

Each 1% increase in parasitemia was associated with a 70% increase in the odds of having at least one WHO severity criterion (aOR = 1.70, 95% CI: 1.40–2.11, p < 0.001). Non–Saudi patients had 2.4 times higher odds of severe malaria compared to Saudi patients (aOR = 2.40, 95% CI: 1.10–5.62, p = 0.027). Healthcare providers should maintain a lower threshold for admission in non–Saudi patients. Age had a neutral effect (p = 0.365), male sex showed a non–significant trend (p = 0.669), and P. vivax infection was not significantly different from P. falciparum (p = 0.216). Both variables directly relate to WHO criteria that define the composite outcome (severe anemia and thrombocytopenia) and including them would introduce circularity. Their strong univariate associations confirm they are important clinical markers of disease severity. **Suppplemtary table S4**

Supplementary Table S4: Univariate and Multivariate Logistic Regression Analysis for Factors Associated with ≥1 WHO Severity Criteria

Variable	Univariate Analysis	Multivariate Analysis	
		aOR (95% CI)	P–value
**Age (per year)**	1.01 (0.99–1.03)	1.01 (0.99–1.04)	0.365

**Sex**			
Female	Reference	Reference	–
Male	1.18 (0.65–2.15)	1.22 (0.50–3.11)	0.669
**Malaria species**			
P. falciparum	Reference	Reference	–
P. vivax	1.35 (0.82–2.23)	1.41 (0.82–2.47)	0.216
**Parasitemia (per 1% increase)**	1.58 (1.35–1.86)	1.70 (1.40–2.11)	<0.001
**Hemoglobin (per g/dL)**	0.85 (0.76–0.95)	—	—
**Platelets (per ×10³/μL)**	0.98 (0.97–0.99)	—	—
**Nationality**	Reference		
Saudi	2.15 (1.12–4.18)	Reference	–
Non–Saudi		2.40 (1.10–5.62)	0.027

— Not included in multivariate model (excluded due to collinearity with outcome definition or not meeting inclusion criteria)

To assess the robustness of our findings, we performed a sensitivity analysis using an alternative definition of severe malaria that excluded hyperparasitemia (>5%) as a criterion (Table S5). The results remained consistent across both definitions. In Model B (excluding hyperparasitemia), parasitemia remained significantly associated with ≥1 WHO criteria (aOR = 1.42, 95% CI: 1.21–1.71, $p < 0.001$), and non–Saudi nationality continued to show increased odds (aOR = 2.48, 95% CI: 1.16–5.71, $p = 0.018$). Age, sex, and malaria species remained non-significant. Model fit was comparable between both definitions. These sensitivity analyses confirm the robustness of our primary findings. **Table S5**

Supplementary Table S5: Multivariate Logistic Regression for ≥1 WHO Severity Criteria (Sensitivity Analysis)

Variable	Model A: Full WHO Definition (including hyperparasitemia)		Model B: Excluding Hyperparasitemia	
	aOR (95% CI)	p–value	aOR (95% CI)	p–value
**Age (per year)**	1.01 (0.99–1.04)	0.365	1.01 (0.99–1.03)	0.416
**Sex (Male vs Female)**	1.22 (0.50–3.11)	0.669	1.12 (0.47–2.76)	0.807
**Malaria species (P. vivax vs P. falciparum)**	1.41 (0.82–2.47)	0.216	1.47 (0.86–2.53)	0.158
**Parasitemia (per 1% increase)**	1.70 (1.40–2.11)	<0.001	1.42 (1.21–1.71)	<0.001
**Nationality (non–Saudi vs Saudi)**	2.40 (1.10–5.62)	0.027	2.48 (1.16–5.71)	0.018

Supplementary figures

Among 311 patients, 19 cases of hyperparasitemia were identified (6.1%). Most cases (79%) were due to P. falciparum, with parasitemia ranging from 6% to 50%, including several above 25%. Only one case (5.3%) involved P. vivax at 6%, which is atypical, and one case (10%) had an unspecified species. Overall, hyperparasitemia was uncommon (≈1 in 16 patients) and predominantly associated with P. falciparum. **Figure 1s**

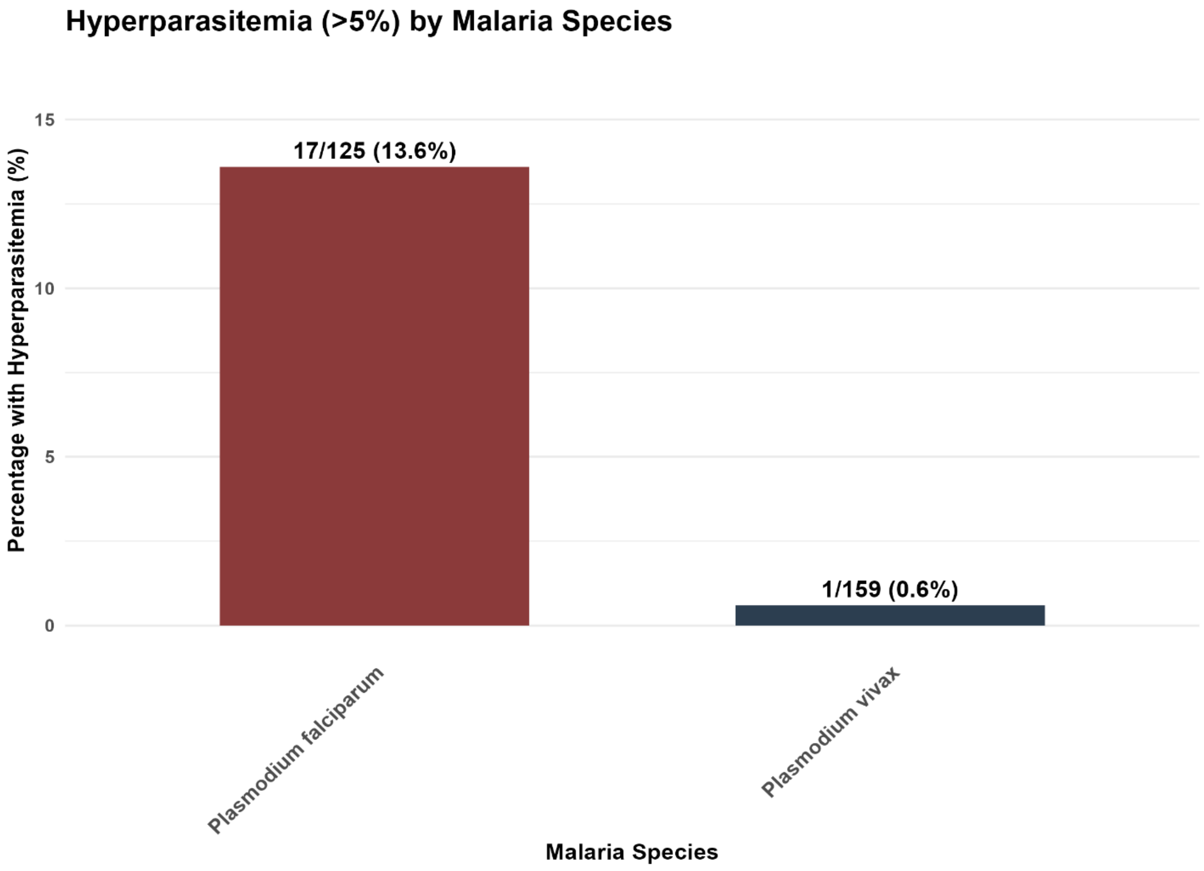

Supplementary Figure S1: Prevalence and Species Distribution of Hyperparasitemia Among Malaria Patients
